# Combining manipulation of integration loci and secretory pathway on expression of an *Aspergillus niger* glucose oxidase gene in *Trichoderma reesei*

**DOI:** 10.1186/s12934-023-02046-w

**Published:** 2023-02-25

**Authors:** Wangli Ji, Xiaolu Wang, Xiaoqing Liu, Yuan Wang, Fangui Liu, Bo Xu, Huiying Luo, Tao Tu, Wei Zhang, Xinxin Xu, Xiaoyun Su

**Affiliations:** 1 Biotechnology Research Institute, Chinese Academy of Agricultural Sciences, No.12 South Zhongguancun St., Haidian District, Beijing, 100081 China; 2 Institute of Animal Sciences, Chinese Academy of Agricultural Sciences, No. 2 West Yuanmingyuan Road, Haidian District, Beijing, 100193 China; 3 College of Biological and Food Engineering, Guangdong University of Petrochemical Technology, Maoming, 525000 Guangdong China

**Keywords:** *Trichoderma reesei*, Glucose oxidase, *Aspergillus niger*, Integration loci, Secretory pathway

## Abstract

*Trichoderma reesei* (*T. reesei*) is well-known for its excellent ability to secret a large quantity of cellulase. However, unlike the endogenous proteins, little is known about the molecular mechanisms governing heterologous protein production. Herein, we focused on the integration loci and the secretory pathway, and investigated their combinatorial effects on heterologous gene expression in *T. reesei* using a glucose oxidase from *Aspergillus niger* as a model protein. Integration in the *cel3c* locus was more efficient than the *cbh1* locus in expressing the *AnGOx* by increasing the transcription of *AnGOx* in the early stage. In addition, we discovered that interruption of the *cel3c* locus has an additional effect by increasing the expression of the secretory pathway component genes. Accordingly, overexpressing three secretory pathway component genes, that were *snc1*, *sso2*, and *rho3*, increased *AnGOx* expression in the *cbh1* transformant but not in the *cel3c* transformant.

**Supplementary Information:**

The online version contains supplementary material available at 10.1186/s12934-023-02046-w.

## Introduction

*Trichoderma reesei* (*T. reesei*) is a well-known filamentous fungus work horse for large scale production of cellulase [1], which has been widely used in food, feed, textile, and biofuel industries. In addition, there is a long history of using *T. reesei* as a host to produce heterologous proteins from bacteria [2], fungi [3], and even mammals [4]. In making *T. reesei* an efficient microbial protein producer, one frequently observed phenomenon is that the initial production level of the heterologous protein is low. For example, eight proteins (including the bovine chymosin, *Phlebia radiate* laccase, *Hormoconis resinae* glucoamylase, Fab antibody fragment, barley endopeptidase B, *Aspergillus niger* acid phosphatase, and *A. niger* lipase) were produced in *T. reesei* to a yield not exceeding 1 g/L (20 ~ 700 mg/L) [5]. This indicates that the host strain and/or the genes of interest require sophisticated engineering toward an ultimately satisfying level of expression, given the fact that the endogenous cellulase can be produced at a level of up to over 100 g/L [1]. As cellulase is the prototype of secretory protein expression in *T. reesei*, the strategies used to improve heterologous gene expression can commonly be reused from those employed in improving cellulase production. Basically, one most applied strategy involves the use of a strong inducible cellulase promoter (in most cases the promoter of *cbh1* encoding cellobiohydrolase I) to direct the transcription of the genes of interest [6].

In cellulase expression, transcription is generally regarded as the major regulatory stage [7]. However, a variety of factors including the selection marker [8], codon bias [3], mRNA stability [9], and other cellular activities including secretion, glycosylation, cell morphogenesis, protease-mediated intra- and extracellular protein degradation, mitochondrial status, and cell metabolism are all able to impact cellulase production. This, in turn, suggests that these regulatory stages can be engineered to improve cellulase production in *T. reesei*. Indeed, the plethora of knowledge has enabled successful improvement of cellulase production [10, 11]. The heterologous genes that can be functionally expressed in *T. reesei* are rapidly expanding in these years [3, 12–16]. Nevertheless, despite these achievements, little is known about whether altering the abovementioned cellular activities such as protein secretion would have effects on heterologous protein production in *T. reesei*. Moreover, much less is known about the effect of combinatorial manipulation of multiple expression-enhancing factors on heterologous protein production. The understanding of combinatorial manipulation is particularly important because strain improvement usually demands multiple and iterative rounds of genetic engineering.

The *Aspergillus niger* (*A. niger*) glucose oxidase (*An*GOx*)* is an enzyme that converts glucose to gluconic acid with concurrent releasing of hydrogen peroxide. This biochemical reaction is of wide application value. For example, *An*GOx is a widely used glucose sensor protein on a variety of chips [17]. In addition, the GOx-catalyzed production of gluconic acid is used in cement to enhance the intensity [18]. Recently, *An*GOx was shown to be beneficial for growth performance of broiler chickens, making it an ideal candidate enzyme in the poultry industry [19]. Previously, we have codon-optimized the coding sequence of *AnGOx*, successfully expressed it in *T. reesei*, and discovered that overexpressing the key component genes (*snc1*, *bip1*, and *hac1*) in the secretion pathway could improve *An*GOx secretion [20]. However, its production level (0.6 U/mL in shake flask fermentation) is still not satisfactory, restricting its use in these areas. This protein has a moderate molecular mass, can be expressed in *T. reesei* (albeit in minor amount), and additionally, has a FAD prosthetic group that needs to be correctly assembled. These collectively make *An*GOx a perfect model to study the mechanisms directing heterologous gene expression in *T. reesei*. Herein, we used *AnGOx* as a model gene to investigate the effects of combining two factors, i.e. the loci of integration (*cbh1* and *cel3c*) and overexpression of the secretory pathway genes (*snc1*, *sso2*, and *rho3*), on expression of a heterologous gene in *T. reesei*.

## Results and discussion

### Expression of *AnGOx* was highly influenced by the integration loci

**Fig. 1:**
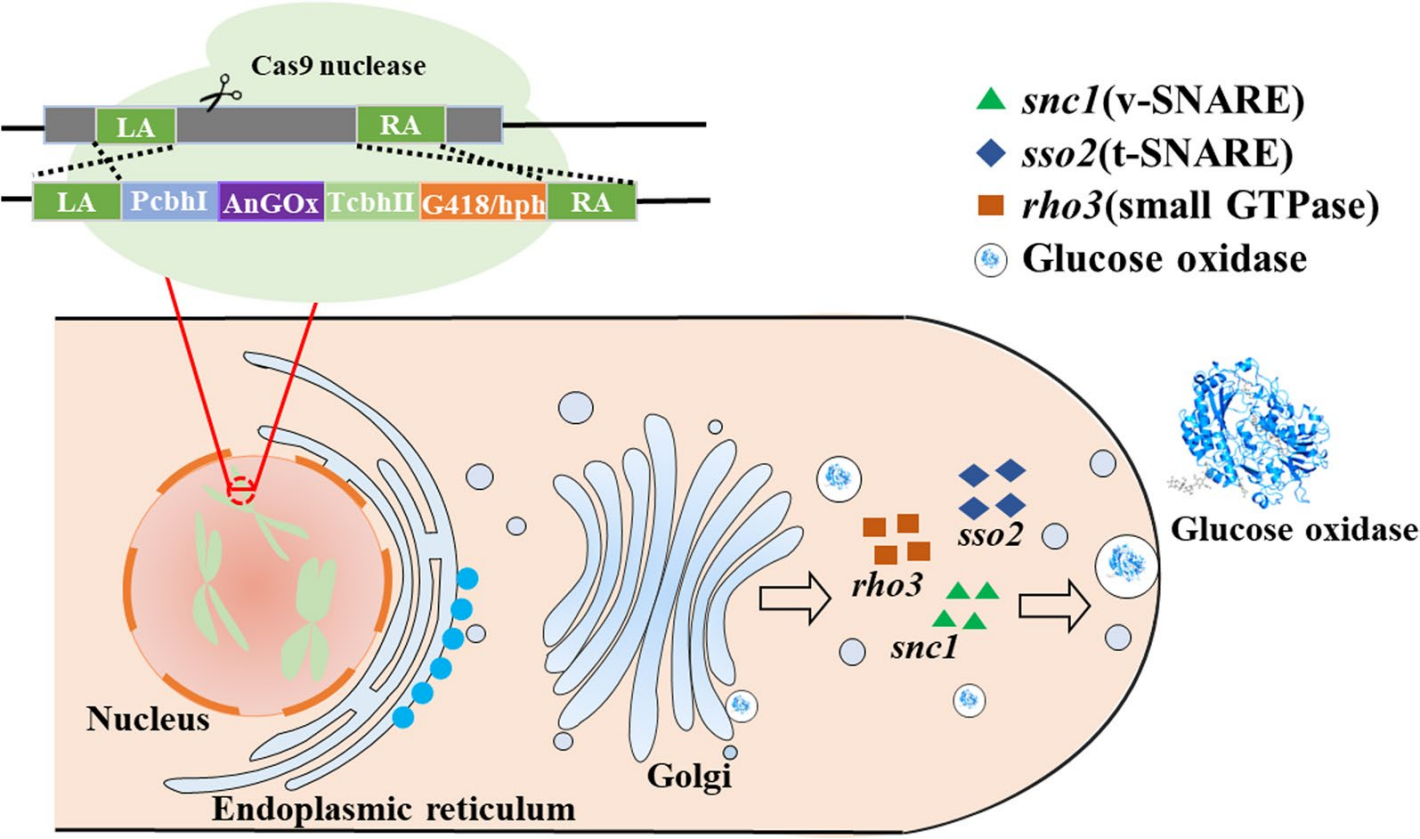
A schematic diagram showing the effects of combining the integration loci and overexpression of selected component genes in the secretion pathway on expression of the *A. niger* glucose oxidase. The investigated integration loci were the *cbh1* and *cel3c* sites, respectively, with the former being a commonly employed one and the latter recently discovered. Three genes, that were *snc1*, *sso2*, and *rho3*, were selected as representative component genes in the secretion pathway for investigation of the effects of their overexpression on secretive *AnGOx* expression. The *snc1* encodes a v-SNARE protein, *sso2* gene encodes a t-SNARE protein, and *rho3* encodes a small GTPase involved in protein secretion

As addressed above, many factors can have profound effects on expression of the heterologous genes. We decided to choose the integration loci and secretory pathway to investigate their individual and combinatorial effects on secretory protein expression (Fig. 1). The reason for choosing the integration loci is that the integration loci are well-known to have a large impact on gene expression, particularly affecting the stage of gene transcription. This is important as cellulase expression is mainly regulated at the transcription level in *T. reesei* [7]. The major, strong cellulase *cbh1* promoter was employed in this study to drive *AnGOx* expression, suggesting that manipulation of the transcription stage could similarly have a significant effect on gene expression. Currently, there are no known self-replicating plasmids in *T. reesei*, indicating that all transformed DNA fragments are integrated into the chromosome. Correspondingly, the integration loci could largely affect gene expression, because the accessibility to transcription factors and RNA polymerase is not identical along the chromosomes [21].

**Fig. 2:**
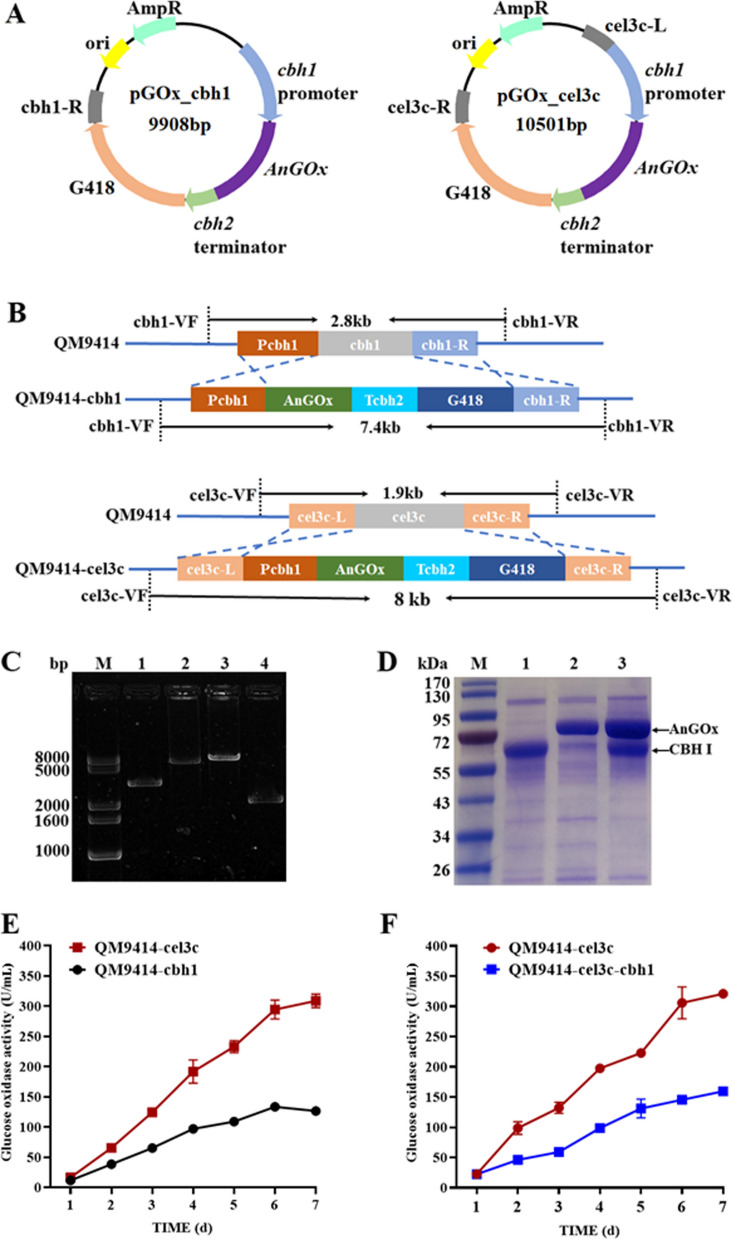
The integration loci were important for expression of *AnGOx* in *T. reesei*. **A**: The plasmid maps of pGOx_cbh1 and pGOx_cel3c. **B**: Schematic diagram showing the DNA fragments that were amplified in diagnostic PCR verification of transformants with integration in the *cbh1* and *cel3c* loci, respectively. The primer pairs used were cbh1-VF/VR and cel3c-VF/VR for integration in *cbh1* and *cel3c*, respectively. **C**. PCR verification of targeted integration of the *AnGOx*-expressing cassette in the *cbh1* and *cel3c* loci. The primer pair cbh1-VF/VR was used for lane 1 and 2, while the primer pair cel3c-VF/VR was used for lane 3 and 4. Lane 1: QM9414; 2: QM9414-cbh1; 3: QM9414-cel3c; 4: QM9414. M: DNA molecular mass marker. **D**: SDS-PAGE of the extracellular proteins of the *T. reesei* parent strain QM9414 and the *cbh1-* and *cel3c*-integrated *AnGOx*-expressing strains. Lane M: protein molecular mass marker; 1: QM9414; 2: QM9414-cbh1; 3:QM9414-cel3c. **E**: Time course analysis of the *An*GOx activity in the supernatant of the *cbh1* (QM9414-cbh1) and *cel3c*-integrated (QM9414-cel3c) *AnGOx*-expressing strains. F: Time course analysis of the *An*GOx activity in the strains of QM9414-cel3c and QM9414-cel3c-cbh1 (with *AnGOx* integrated in both *cbh1* and *cel3c*) strains

Random integration was used in our previous overexpression of *AnGOx* in *T. reesei*, raising the uncertainty to the final yield of *An*GOx. Therefore, to minimize the uncertain effect of integration loci on gene expression, targeted integration of *AnGOx* into two genetic loci (*cbh1* and *cel3c*) was employed. These two loci were either well-known (*cbh1*) or newly identified (*cel3c*, which encodes a β-glucosidase) [12], likely favoring heterologous gene expression. In *T. reesei*, *cbh1* is the most favored locus because *cbh1* is induced up to several thousand folds on the cellulose culture medium [7], suggesting that the chromosome of this locus must be in a less condensed state in the induction state. Although other loci favorable for protein production have been discovered, no report was available for a comparison of these different sites. Therefore, *AnGOx* was targeted to one of the two loci (*cbh1* and *cel3c*) for heterologous expression in *T. reesei*. Correct integration of *AnGOx* using the DNA fragments amplified from pGOx_cbh1 and pGOx_cel3c (Fig. 2A) in these loci was verified by diagnostic PCR (Fig. 2B), as demonstrated by a 7.4-kbp and a 8.0-kbp DNA fragment in the *AnGOx* transformants targeting to *cbh1* and *cel3c*, respectively. In contrast, the same primer pairs amplified 2.8-kbp and 1.9-kbp in the parental strain QM9414 (Fig. 2C). In addition, a single copy of *AnGOx* was detected in both strains as analyzed by quantitative PCR (data not shown).

Interestingly, *AnGOx* was expressed to a higher level when integrated in the *cel3c* locus than that in the *cbh1* site, as demonstrated by SDS-PAGE analysis and assay of the GOx activity. On day 7 post cellulose induction, *AnGOx* was expressed to a higher level, which could be easily visualized on the SDS-PAGE gel (Fig. 2D). The glucose oxidase activity was 309 U/mL in the *cel3c*-integrated site and 126 U/mL in the *cbh1*-integrated site (Fig. 2E). In *T. reesei*, CBH1 is the major extracellular protein, accounting to up to 60% of the total extracellular proteins. Integration at the *cbh1* locus disrupted synthesis of the CBH1 protein, thereby likely releasing the secreting capacity of the cell available for other proteins such as the heterologous *An*GOx. Therefore, the comparably lower expression for the *cbh1*-integrated expression was somewhat unexpected and suggested that there might be unveiled mechanisms governing the *cel3c-*integrated expression. Moreover, simultaneous integration of one copy of the *AnGOx* gene in the *cbh1* and *cel3c* loci, respectively, did not increase its expression. On the contrary, the extracellular GOx activity reached to a level (160 U/mL) slightly higher than that obtained with integration to *cbh1* (Fig. 2F).

### *AnGOx* was transcribed at a higher level in the *cel3c* locus at the early stage of induction

**Fig. 3:**
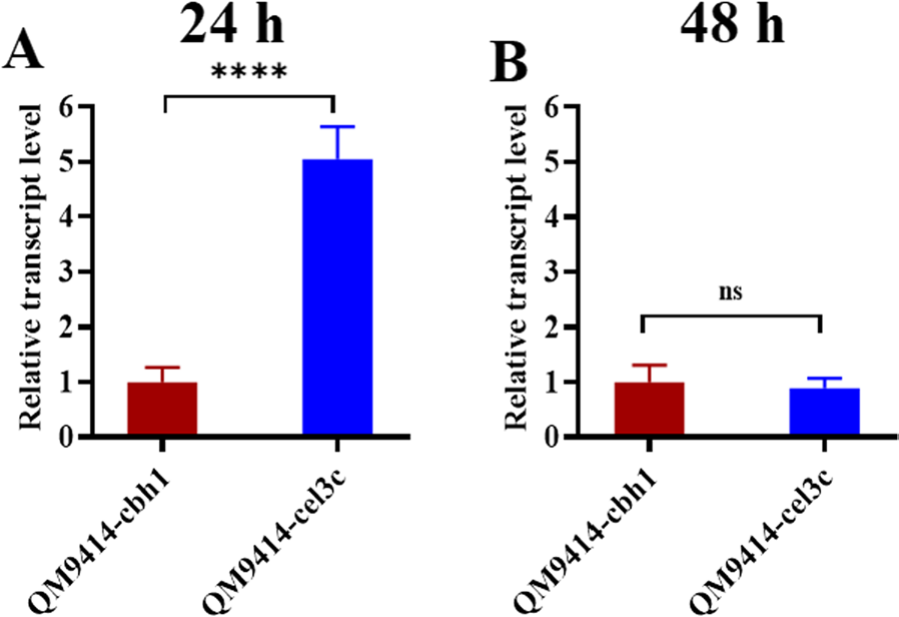
*AnGOx* was expressed at a higher level in the early stage of induction in QM9414-cel3c transformant as analyzed by RT-qPCR. **A** and **B**: comparison of the *AnGOx* expression in QM9414-cbh1 and QM9414-cel3c strains at 24 h (**A**) and 48 h **B**. The *T. reesei* cells were induced with cellulose. At 24 h and 48 h post induction, mycelia were collected. The total RNA was extracted from the mycelia and then reverse-transcribed to cDNA. In RT-qPCR analysis, the *sar1* gene was used as the internal reference gene. For both the 2 time points, the transcript abundance of *AnGOx* in the QM9414-cbh1 was set as 1.0. The relative transcript level of *AnGOx* in the QM9414-cel3c was calculated through dividing by that in the QM9414-cbh1 strain. *ns* not significant; ****, *P* < 0.0001

The transcript level of *AnGOx* in the *cbh1*-integrated transformant was compared to that in the *cel3c*-integrated strain. RT-qPCR analysis of the two strains cultivated on cellulose-containing inducing medium indicated that the transcript level of *AnGOx* in the *cel3c* transformant was 5.0-fold higher than that in the *cbh1* transformant at 24 h post induction (Fig. 3A). However, with the culture continuing, the difference between the *AnGOx* transcript levels in the two strains was diminished: the relative transcript level of *AnGOx* in the *cel3c*-integrated strain was 0.89-fold that of the *cbh1*-integrated strain at 48 h post induction (Fig. 3B). The *cel3c* transcript level is not high in *T. reesei* [22]. However, as the observations by us and other researchers were clearly suggestive of the ability of the *cel3c* locus to drive robust transcription, it was assumed that the *cel3c* gene transcript might have a short half-life [12]. In addition, because the *cbh1* promoter used in this study is as long as 1.5-kb, the effect of the upstream sequence on *AnGOx*-expression may not be as significant as that described for a hybrid promoter (constructed by fusing core regions of the Pcdna1 and Pcbh1 [6]. There have been studies indicating that specific loci with a more open chromosome state are responsible for the more favorable heterologous gene expression [23]. This suggested that the *cel3c* locus in the chromosome might also have such an open state at the early stage of induction. However, it was also noticed that, while the difference of the transcription levels between the two strains was diminishing, the difference in the extracellular *An*GOx enzyme level was growing larger. This strongly suggested that integration in the *cel3c* locus might have an additional regulatory role for expression of the heterologous *AnGOx*.

### The combinatorial effects of overexpressing component genes in the secretory pathway on *AnGOx* expression

In addition, another factor, i.e. the secretory pathway, was also selected for investigation solely or in combination with the integration loci. In many microbial organisms, overexpressing the heterologous genes is known to incur high burdens to many facets of the cellular activities [24–26]. One most frequently observed phenomenon is the competition of endogenous and heterologous proteins for the protein folding apparatuses, which can be alleviated by introducing extra copies of folding chaperones [27]. Heterologous gene expression can also impose a similarly heavy burden to the protein translocation process to the extracellular milieu, although this process is much less known in *T. reesei*. Specifically, *An*GOx expression can be improved by overexpressing key component genes in the secretory pathway. Therefore, the integration loci and manipulation of the secretory pathway were selected as two representative factors to investigate their impacts on expression of *AnGOx* (Fig. 1).

**Fig. 4:**
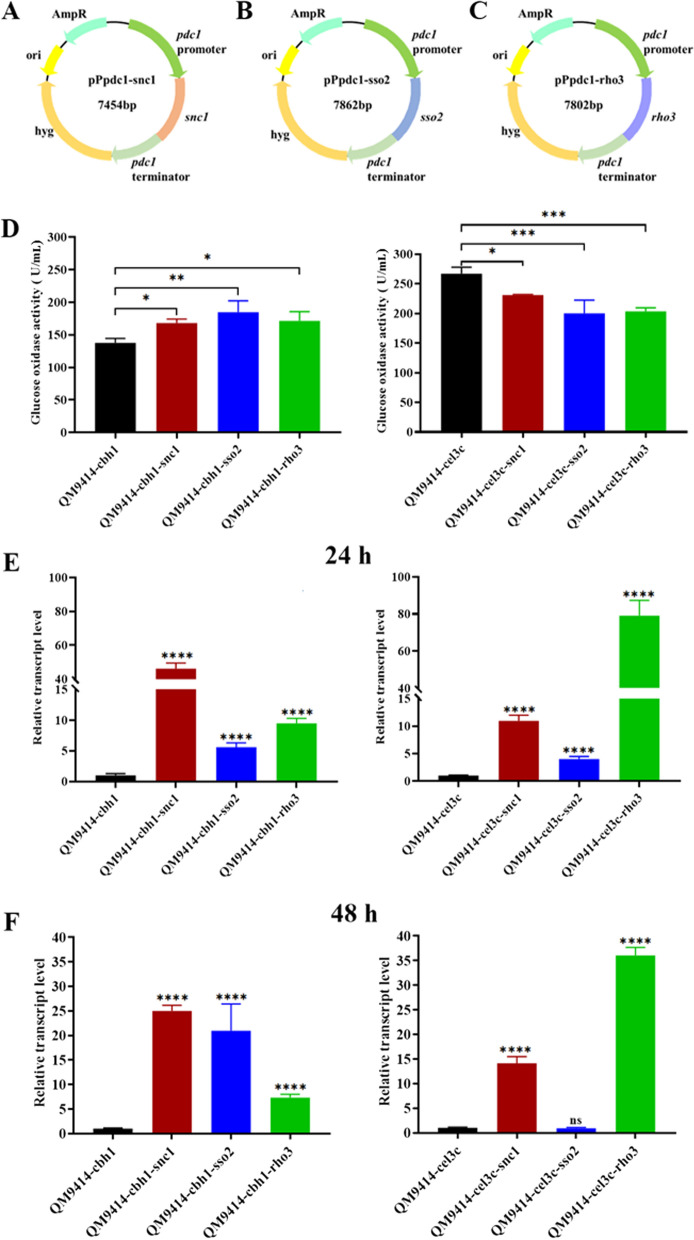
Effect of overexpressing three component genes in the secretion pathway on the secretory production of *An*GOx in *cbh1*-integrated and *cel3c-*integrated strains. A-C: The plasmid maps for pPpdc1-snc1 (**A**), pPpdc1-sso2 (**B**) and pPpdc1-rho3 (**C**). **D**: The glucose oxidase activities in QM9414-cbh1, QM9414-cbh1-snc1, QM9414-cbh1-sso2, QM9414-cbh1-rho3, QM9414-cel3c, QM9414-cel3c-snc1, QM9414-cel3c-sso2, and QM9414-cel3c-rho3 on day 6 post induction. E and F: The relative transcript levels of *snc1* in QM9414-cbh1-snc1 (QM9414-cbh1 overexpressing the secretory component gene *snc1*), *sso2* in QM9414-cbh1-sso2, *rho3* in QM9414-cbh1-rho3, *snc1* in QM9414-cel3c-snc1 (QM9414-cel3c overexpressing the secretory component gene *snc1*), *sso2* in QM9414-cel3c-sso2, and *rho3* in QM9414-cel3c-rho3 at 24 h (**E**) and 48 h (**F**) post cellulose induction. The transcript level of the corresponding genes in QM9414-cbh1 or QM9414-cel3c was set as the reference with a value of 1.0. The relative transcript levels of the genes in the other strains were calculated through dividing by the corresponding genes in the QM9414-cbh1 or QM9414-cel3c strains. *, 0.01 < *P* < *0.05*; **, *0.001* < *P* < *0.01*; ***, *P* < *0.001*

The DNA fragments for overexpressing three different component genes (*snc1*, *sso2*, and *rho3*) amplified form the pPpdc1-snc1 (Fig. 4A), pPpdc1-sso2 (Fig. 4B), and pPpdc1-rho3 (Fig. 4C) plasmids in the secretory pathway were individually transformed into *cbh1*- and *cel3c*-integrated transformants. The *snc1* gene encodes a v-SNARE protein and has been already demonstrated to be able to improve *AnGOx* secretion in *T. reesei* [20]. *sso2* encode t-SNARE proteins involved in vesicle fusion to the plasma membrane and *rho3* codes for a *ras*-type small GTPase involved in cell polarity and vesicle fusion with the plasma membrane [28]. In the *cbh1*-transformant, all three component genes were able to improve *AnGOx* expression significantly. On day 6 post induction, while the GOx activity was 137 U/mL in the QM9414 parent strain, the activity reached to 168, 185, and 171 U/mL for the representative *snc1-*, *sso2-*, and *rho3-* transformants (Fig. 4D). However, when these genes were overexpressed in the *cel3c-*integrated transformant, no improvement was observed for any of the three genes (Fig. 4D). An RT-qPCR analysis indicated that at 24 h and 48 h post cellulose induction, the transcripts of all three genes (*snc1*, *sso2*, and *rho3*) were significantly increased due to the overexpression except that of *sso2* in the QM9414-cel3c strain at 48 h (Fig. 4E and F).

### *AnGOx* integration in *cel3c* increased gene expression in the secretory pathway

**Fig. 5:**
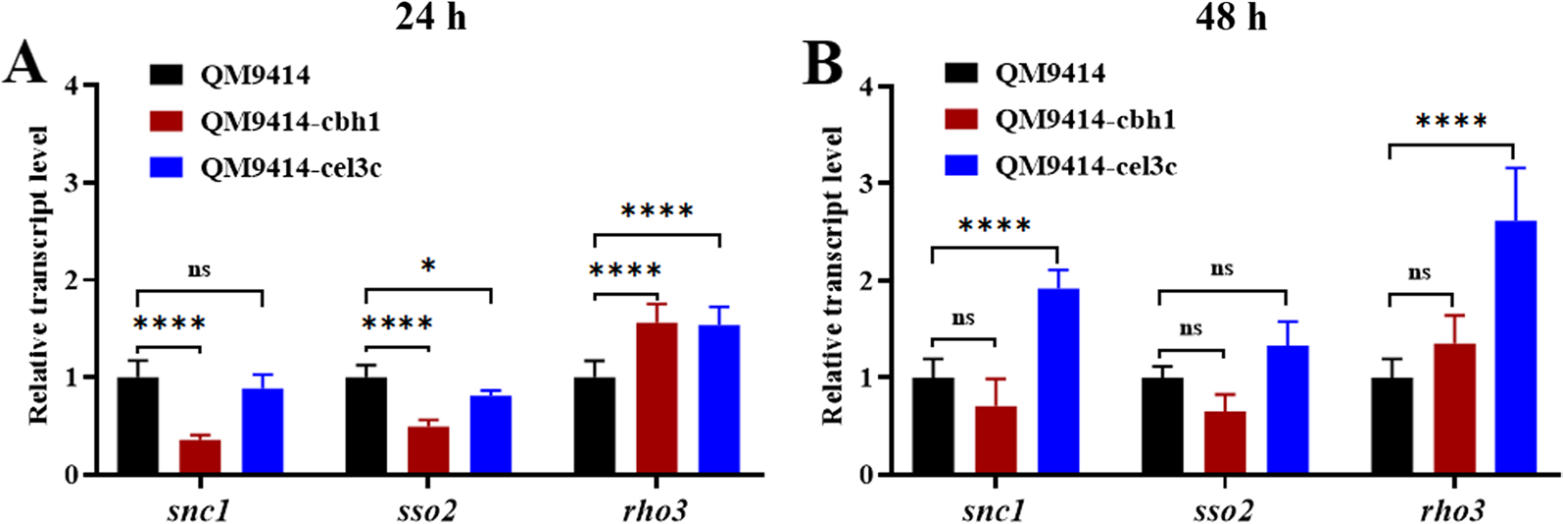
RT-qPCR analysis of the three secretory pathway component genes in the *cbh1*-integrated and *cel3c-*integrated *T. reesei* strains. **A**: Analysis of the transcription for samples taken at 24 h post induction; **B**: Analysis of the transcription for samples taken at 48 h post induction. Expression of *AnGOx* was induced with cellulose in these *T. reesei* strains. At 24 h and 48 h post induction, mycelia were collected. The total RNA was extracted from the mycelia and then reverse-transcribed to cDNA. In RT-qPCR analysis, the *sar1* gene was used as the internal reference gene. For both the 2 time points and all three genes, the transcript abundance of the analyzed gene in the QM9414 was set as 1.0. The relative transcript level of the gene in QM9414-cbh1 and QM9414-cel3c were calculated through dividing by that in the QM9414 strain. *ns* not significant; *, 0.01 < *P* < *0.05*; ****, *P* < *0.0001*

The higher transcription level of *AnGOx* in the *cel3c* locus, plus the inability of improving *AnGOx* expression by overexpressing the key component genes in the secretory pathway in the *cel3c* transformant, collectively pointed out to the hypothesis that the secretory pathway was altered when the *AnGOx* gene was inserted into the *cel3c* locus. Therefore, an RT-qPCR analysis was further used to determine the transcription of the above three selected secretory pathway component genes. At 24 h post cellulose induction, in comparison with QM9414, the transcription of *snc1* and *sso2* in QM9414-cbh1 was significantly decreased, while that in QM9414-cel3c remained nearly unchanged. The transcription of *rho3* was almost identical in both strains, 1.5-fold higher than that in QM9414 (Fig. 5A). However, at 48 h, while those of *snc1*, *sso3*, and *rho3* in QM9414-cbh1 were still lower or comparable to that in QM9414, those of *snc1* and *rho3* in QM9414-cel3c were 1.9- and 2.6-fold, higher respectively (Fig. 5B).

The results undoubtedly indicated that integration of a heterologous gene in different loci can lead to much different expression levels. It is also suggested that the integration can significantly alter cellular activities of the cell physiology. In the case of *AnGOx*, integration in the *cel3c* locus appeared to be both more favorable for transcription at the early induction stage and enhance the secretory pathway machinery, which together contribute to the higher expression of *An*GOx in the *cel3c* transformant. The current finding sheds light on iterative engineering of *T. reesei* for efficient expression of heterologous genes, pinpointing to the complex effects of integration loci on heterologous gene expression and the need to deliberately select the candidate loci for maximal transcription and secretion. Although in this study we only used the model *AnGOx* gene, it was expected that, for other heterologous genes, a similar strategy can also be applied. It was suggested that more integration loci, more component genes in the secretory pathway, and maybe key genes in other regulatory stages can be tested towards high efficiency in stimulating expression of the gene of interest.

## Conclusions

Two important factors, i.e. the integration locus and the secretory pathway, were chosen in this study for investigation of their effects on expression of a heterologous gene in the filamentous fungus workhorse *T. reesei*. Using *AnGOx* as a model heterologous gene, it was demonstrated that integration in the *cel3c* locus was more efficient in transcription of the heterologous gene at the early stage of induction, generating 309 U/mL of *An*GOx in the culture supernatant. Integration in the *cel3c* locus appeared to also potentiate the secretion ability of *T. reesei*, as expression of the secretory component genes *snc1* and *rho3* was elevated. Further overexpressing these secretory component genes improved *AnGOx* expression in the *cbh1* transformant but not in the *cel3c* strain.

## Materials and methods

### Strains and culture conditions

The *Escherichia coli* Trans-T1 strain was used for plasmid construction, propagation, and maintenance in this study. The *A. niger* ZJ5 strain bearing the glucose oxidase gene was isolated from a forest soil sample, which was collected in the Yunnan Province of China. The *T. reesei* strain QM9414 (ATCC 26,921) was used in this study to express *AnGOx*. The QM9414 and its transformants were maintained on potato dextrose agar (PDA) plates at 28 °C for sporulation. Harvested spores were inoculated in the liquid minimal medium (MM, containing (NH_4_)_2_SO_4_, 5.0 g/L; KH_2_PO_4_, 15 g/L; MgSO_4_, 0.6 g/L; CaCl_2_, 0.6 g/L; FeSO_4_·7H_2_O, 0.005 g/L; MnSO_4_·H_2_O, 0.0016 g/L; ZnSO_4_·7H_2_O, 0.0014 g/L; CoCl_2_, 0.002 g/L) supplemented with 2% glucose and 0.5% wheat bran for mycelial growth.

### Plasmid construction

To construct the plasmid (pGOx_cbh1) for targeted integration of the *AnGOx*-expresing cassette in the *cbh1* locus, The 1788-bp *AnGOx* gene fragment was amplified by polymerase chain reaction (PCR) from the genomic DNA of *A. niger* ZJ5 with the primer pair GOx-F/R, while a 600-bp *cbh2* terminator fragment was amplified from *T. reesei* QM9414 using the primer pair Tcbh2-F/R. All primer sequences were listed in Additional file 1: Table S1. The two DNA fragments were in vitro fused together to obtain a 2388-bp fragment by overlap extension PCR with the primers GOD-F and Tcbh2-R. A 600-bp *cbh1* fragment was amplified from the genomic DNA of QM9414 by using the primer pair cbh1R-F/R and a 2471-bp DNA fragment containing the expression cassette of neomycin resistance gene for conferring G418 resistance was amplified from pM13-G418 using the primers G418-F/R [29]. The two DNA fragments were fused into a 3071-bp fragment via overlap extension PCR with the primers G418-F/cbh1R-R. The primers Pcbh1-F and Pcbh1-R were used to amplify a 1551 bp *cbh1* promoter (Pcbh1) fragment from QM9414. The 2898 bp M13 fragment was amplified from pBlueScript-SK cloning vector with the primers M13-F and M13-R. The four fragments were mixed, and the entire 9908 bp recombinant plasmid (pGOx_cbh1) was assembled by using the One Step Cloning Kit of Vazyme (Nanjing, China) utilizing a Gibson assembly method. The other two plasmids (pGOx_cel3c and pGOx_cel3c_hyg) for targeted integration of the *AnGOx*-expressing cassette in the *cel3c* locus were similarly constructed.

The CRISPR/Cas9-mediated genome editing was used for targeted integration of the *AnGOx*-expressing cassette into one of the two selected genomic loci. In this process, two DNA fragments were used, with one for expressing a functional Cas9 and one for expressing a sgRNA targeting one of the specific locus. For the first DNA fragment, the 5323-bp *Cas9*-expression cassette from *Streptococcus pyogenes* was amplified from the pLC2 plasmid with the primers Cas9-F/R [30] for transformation. To construct the DNA fragment that specifically for integration in the *cbh1* locus. Firstly, the small guide RNA (sgRNA) of *cbh1* locus was predicted by http://chopchop.cbu.uib.no/, with the sequence 5ʹ GTCGGCCTGCACTCTCCAAT 3ʹ. Next, the primers sgRNASPF/R were used to amplify a 420-bp PU6-sgRNA(cbh1) fragment from pLC2, and the primers sgRNAcbhISSF/R were used to amplify a 96-bp sgRNA(cbh1)-scaffold from pLC2. Then, the two DNA fragments were fused to a 496-bp DNA fragment by overlap extension PCR with the primers sgRNASPF and sgRNASSR. Finally, for construction of pBlunt-sgRNA(cbh1), the sgRNAcbh1 fragment and Blunt Simple Cloning Vector were combined by using pEASY-Blunt Simple Cloning Kit of TransGen Biotech (Beijing, China). After sequencing correctly, sgRNASPF and sgRNASSR primers were used to amplify sgRNA(cbh1) for transformation. The same method was applied for prediction sgRNA (5ʹ- GTCATCCAGTCCTTCCATCA-3ʹ) of *cel3c* locus and construction of the sgRNA(cel3c) for targeting the cel3c locus.

The plasmids pPpdc1-snc1, pPpdc1-sso2, and pPpdc1-rho3 were used for overexpression of the three genes (*snc1*, *sso2*, and *rho3*) involved in the secretory pathway. For construction of pPpdc1-snc1, the *pdc1* promoter, *snc1* gene, and the *pdc1* terminator were amplified from the genomic DNA of QM9414 using the primer pairs (*pdc1* promoter: Ppdc1-F/R; snc1: snc1-F/R; pdc1 terminator: Tpdc1-F/R). The *hph* gene conferring hygromycin resistance was amplified from the pLC2 plasmid with the primers hyg(s)-F/R. Then these four fragments were mixed together and assembled into the *Not*I and *Xho*I restriction sites of pBlueScript-SK using the Gibson assembly method to finally generate the plasmid pPdc1-snc1. The other two plasmids were similarly constructed.

### Targeted integration of *AnGOx* into the two genomic loci in *T. reesei*

The expression cassette of *AnGOx* was introduced into *T. reesei* by protoplast transformation. Transformation of *T. reesei* was carried out following the method described by Penttilla et al. with some modifications [31]. Briefly, freshly harvested spores of *T. reesei* were grown on PDA plates at 28 °C for 14 h. Then the mycelia were collected, washed with sterilized H_2_O, and treated with 10 mg/mL of Lysing Enzymes (L1412, Sigma-Aldrich, St. Louis, MO) plus 1 mg/mL of cellulase (ONOZUKAR-10). The incubation was continued at 30 °C and terminated when large amounts of protoplasts were released. The *AnGOx*-G418-expressing cassette, sgRNA fragment, and Cas9-expressing cassette were amplified from the plasmids pGOx_cbh1, pBlunt-sgRNA(cbh1) and pLC2 using the primers Pcbh1-F/cbhR-R sgRNASPF/SSR, and Cas9-F/R, respectively, and 10 μg each of the DNA fragments were mixed and co-transformed into *T. reesei* protoplasts. The transformants were selected on PDA agar plates supplemented with 100 μg/mL G418 for 3 d. Colonies were picked out and inoculated on PDA plates containing the corresponding antibiotics for further selection. PCR was used to verify whether the target gene was integrated into the *cbh1*, and cel3c sites with the primer pairs cbh1-VF/R and cel3c-VF/R, respectively. The same method was used to integrate the *AnGOx*-hyg-expressing cassette into the *cel3c* locus of strain QM9414-cbh1.

### Overexpression of component genes in the secretory pathways

The QM9414-cbh1 and QM9414-cel3c strains (bearing targeted integration of *AnGOx* in the *cbh1* and *cel3c*, respectively) were used for protoplast preparation. The protoplasts were transformed with 10 g of the DNA fragments containing each of the expressing cassettes for *snc1*, *sso2*, and *rho3* (obtained by PCR with the primers Ppdc1-F and hyg(s)-R) and the transformants were spread and selected on PDA plates supplemented with 50 μg/mL hygromycin. Integration of the genes encoding the secretory pathway component proteins in the fungal chromosome was verified by PCR using the paired primers VF(snc1)/VR(hyg), VF(sso2)/VR(hyg) and VF(rho3)/VR(hyg), respectively.

### Induction of *AnGOx* expression

The *T. reesei* strains transformed with the *AnGOx* gene were cultured on PDA plates for sporulation. Then freshly harvested spores (1 × 10^6^/mL) were inoculated into the MM medium supplemented with 2% glucose and 0.5% wheat bran for mycelial growth. Mycelia were washed with distilled water and then transferred to MM plus 2% Avicel and 1.4% gluconic acid (a compound that can increase release of glucose oxidase into the fermentation broth) for *An*GOx induction [32]. All cultures were carried out at 28 °C.

### Assay of GOx activity

The glucose oxidase activity was determined according to the method described by Gao et al. [33]. Briefly, the reaction mixture containing 341 μM *o*-dianisidine in 2.5 mL 100 mM NaH_2_PO_4_/Na_2_HPO_4_ buffer (pH 6.0), 300 μl 18% (w/v) D-glucose, and 100 μl horseradish peroxidase (90 U/mL) were pre-heated at 30 °C for 2 min. Then, 100 μl enzyme was added and the system was incubated at 30 °C for 3 min. The reaction was terminated by the addition of 2 mL of 2 M sulfuric acid and determination of absorbance value at 540 nm. One unit of glucose oxidase activity (U) was defined as the enzyme required to transform 1 mol of D-glucose into gluconic acid and H_2_O_2_ in 1 min at 30 °C and pH 6.0.

### Reverse transcription quantitative PCR

The transcript levels of *AnGOx* and the secretory component genes were measured by reverse transcription quantitative PCR (RT-qPCR) in a QuantStudio 6 Flex System. At 24 h and 48 h post induction, mycelia were collected for total RNA extraction. The mycelia were pulverized using a Mini-Beadbeater (BioSpec, Bartlesville, OK) and the total RNA was extracted by using the TRIzol reagent (Thermo Fisher Scientific, Waltham, MA). The total RNA (1 μg) was treated with DNase I and then reverse-transcribed to cDNA using the First Strand cDNA Maxima Synthesis kit (TOYOBO, Shanghai, China). RT-qPCR was performed in a QuantStudio 6 Flex Real-Time PCR System (Applied Biosystems, San Diego, CA) using a TransScript Green One-Step qRT-PCR SuperMix (TransGen, Beijing, China). The *sar1* gene [34] was used as a reference. The primers in RT-qPCR were listed in Additional file 1: Table S1. The following PCR procedure was used: initial denaturation at 95 ℃ for 10 min and then 40 cycles of 94 ℃ for 10 s, 60 ℃ for 20 s, and 72 ℃ for 30 s. The transcript level of each gene was estimated by using the 2^–*ΔΔ*Ct^ method [35]. Briefly, the *Δ*Ct value of each gene was calculated by subtracting the Ct value of the *sar1* gene from those of the tested genes (*Δ*Ct = Ct_tested_ − Ct_sar1_). The *ΔΔ*Ct value of each gene was calculated by subtracting the *Δ*Ct value of the parent stain from that in the transformant stain (*ΔΔ*Ct = *Δ*Ct (of the transformant) − *Δ*Ct (of the parent).

### Statistical analysis

All of the assays were carried out in triplicate. Statistical significance of the transcript levels was determined by *t* test analysis using the Prism GraphPad software (Insightful Science, San Diego, CA). For other data, one-way analysis of variance (ANOVA) was carried out to determine the significance in difference. Asterisks were used to indicate significant differences: *, 0.01 < *P* < 0.05; **, 0.001 < *P* < 0.01; ***, *P* < 0.001; ****, *P* < 0.0001; ns, not significant.

## Supplementary Information

**Additional file 1**: **Table S1**. Primers used in this study.

## Data Availability

All of the data generated in this study are available and have been already included in the main text and supplemental material.
