# Supplementary material for: Combining manipulation of integration loci and secretory pathway on expression of an Aspergillus niger glucose oxidase gene in Trichoderma reesei
Source: Microb Cell Fact. 2023 Feb 25;22:38. doi: 10.1186/s12934-023-02046-w (PMC9960163; doi:10.1186/s12934-023-02046-w)
Supplement: Supplementary file 1 — Additional file 1: Table S1. Primers used in this study. [file 12934_2023_2046_MOESM1_ESM.docx]

**Additional file: Table S1. Primers used in this study**

| **Primer** | **Sequence (5’-3’)** | **Usage** |
| --- | --- | --- |
| Pcbh1-F | TATCTAGAGTTGTGAAGTCGGTAATCCCGC | Amplification of the *cbh1* promoter |
| Pcbh1-R | CATTGCTCCTGATGTAGTGTGGGAGAGCACGAGCTGTGGCCAAGAAGGCCGAGATG |  |
| GOx-F | CACAGCTCGTGCTCTCCCACACTACATCAGGAGCAATGG | Amplification of the *AnGOx* gene |
| GOx-R | GGTCACGAAAGCCTCAATGGTGATGGTGATGGTGCTGCATAGAAGCGTAATCCGCCAAG |  |
| Tcbh2-F | ACGCTTCTATGCAGCACCATCACCATCACCATTGAGGCTTTCGTGACCGGGCTTCAAAC | Amplification of the *cbh2* terminator |
| Tcbh2-R | GCAACCGCGGCTTTCTATTCTGCATTAAC |  |
| G418-F | TAATGCAGAATAGAAAGCCGCGGTTGCGGATCCCCCGACTAGTGCGCGATCGC | Amplification of the G418-resistance gene |
| G418-R | GCTGTTACCGCTCGTGGTAACCGGTGTCTCGCACACGGCTTCGACGGCGTTTC |  |
| cbh1R-F | GTGCGAGACACCGGTTACCACGAGCGGTAACAGCCTCTC | Amplification of the cbh1R |
| cbh1R-R | ATCGAGGGTAAAGCTTGAGCCAGGGC |  |
| M13-F | CTGGCTCAAGCTTTACCCTCGATCTCGAGGGGGGGCCCGGTACCCAGCTT | Amplification of the M13 plasmid |
| M13-R | TACCGACTTCACAACTCTAGATAGCGGCCGCCACCGCGGTGGAGCTCCAATT |  |
| Cas9-F | GCATGCGGAGAGACGGACGGACGCAGAGAGAA | Amplification of the *cas9* expression cassette |
| Cas9-R | CATGCATTGCAGATGAGCTGTATCTGGAAGAGG |  |
| sgRNASPF | CCCAGTAGCGAAAATGCCACTCCAGAC | Amplification of the sgRNA expression cassette |
| sgRNASSR | GCACCGACTCGGTGCCACTTTTTCAAG |  |
| sgRNAcbh1SPR | CTAAAACATTGGAGAGTGCAGGCCGACACTTGTGAAGACATTAGAATGAACT | Amplification of the sgRNAcbh1 |
| sgRNAcbh1SSF | CACAAGTGTCGGCCTGCACTCTCCAATGTTTTAGAGCTAGAAATAGCAAGTT |  |
| cbh1-VF | gtgggactttgatggtcatcaaacaaagaacgaag | Verification of *AnGOx* integration in the *cbh1* locus |
| cbh1-VR | CCTTCTTGAACTGAGTCAGGCCGCCCTTGTCTGAG |  |
| cel3cL-F | TAGGGCGAATTGGAGCTCCACCGCGGTGGCCGGCTTAATGCCGGTTAAAGC | Amplification of the cel3cL |
| cel3cL-R | GCGGGATTACCGACTTCACAACTCTAGATAAAGGGTCGGGTGAATAGAACTC |  |
| cel3cR-F | AACGCCGTCGAAGCCGTGTGCGAGACACCGCTTCCCTTGCGGCACGTC | Amplification of the cel3cR |
| cel3cR-R | ACAAAAGCTGGGTACCGGGCCCCCCCTCGATTTCCGTTGGAGACGTTGAAC |  |
| sgRNAcel3cSPR | AAAACTGATGGAAGGACTGGATGACACTTGTGAAGACATTAGAAT | Amplification of the sgRNAcel3c |
| sgRNAcel3cSSF | CAAGTGTCATCCAGTCCTTCCATCAGTTTTAGAGCTAGAAATAGC |  |
| cel3c-VF | CTTGTGTGGAGTTGGACCCCGATCTTCTCCAATTC | Verification of *AnGOx* integration in the *cel3c* locus |
| cel3c-VR | CAAAGGGAGTGACTGCGTAGTAGGCCCTGAGTGCGG |  |
| hyg-F | GTTAATGCAGAATAGAAAGCCGCGGTTGCTCGACAGAAGATGATATTGAAGGAGCAC | Amplification of the *hph*-resistance gene |
| hyg-R | AAGAAGGATTACCTCTAAACAAGTGTACCTGTGC |  |
| snc1-F | CAAAGCAGCGCAGCTACAGCACAATCATGGCCGACGCTCCG | Amplification of the *snc1* gene |
| snc1-R | CTACCCGGTCAGACTTCATGCCGGGTTAACGGGTGGCAACG |  |
| sso2-F | CAAAGCAGCGCAGCTACAGCACAATCATGGCTCCTTTGACG | Amplification of the *sso2* gene |
| sso2-R | CTACCCGGTCAGACTTCATGCCGGGAACCAACATACCAACA |  |
| rho3-F | CAAAGCAGCGCAGCTACAGCACAATCATGCCTCTCTGCGGC | Amplification of the *rho3* gene |
| rho3-R | CTACCCGGTCAGACTTCATGCCGGGTTACATGATTGTGCACT |  |
| Tpdc1-F | CCCGGCATGAAGTCTGACCGGGTAG | Amplification of the *pdc1* terminator |
| Tpdc1-R | CGTCGGCCGGGTGGTGAGCTTCTGA |  |
| Ppdc1-F | GGAGCTCCACCGCGGTGGCGGCCGCGTGTCGAGCCGGGAGGAGTTTTCGC | Amplification of the pdc*1* promoter |
| Ppdc1-R | GATTGTGCTGTAGCTGCGCTGCTTTG |  |
| hyg(s)-F | GTCAGAAGCTCACCACCCGGCCGACGTCGACAGAAGATGATATTGAAGGAGC | Amplification of the *hph*-resistance gene |
| hyg(s)-R | GCTGGGTACCGGGCCCCCCCTCGAGAAGAAGGATTACCTCTAAACAAGTG |  |
| VR(hyg) | GTCCTCGTTCCTGTCTGCTAATAAG | Verification of *sso1* integration in the chromosome |
| VF(sso2) | CATTGTTCTCATCTACATCTTCGTC | Verification of *sso2* integration in the chromosome |
| VF(snc1) | GACATGAAGATGCGCGTCTGCATCG | Verification of *snc1* integration in the chromosome |
| VF(rho3) | CACAGATGACACCGATTTGATCGTGC | Verification of *rho3* integration in the chromosome |
| GOx-F(qPCR) | CAGAGGTCCTATCATTGAGGAC | Amplification of the *AnGOx* gene for qPCR |
| GOx-R(qPCR) | GTCTCCCATGAGTCAACTTGTG |  |
| sar1-F(qPCR) | TGGATCGTCAACTGGTTCTACGA | Amplification of the *sar1* gene for qPCR |
| sar1-R(qPCR) | GCATGTGTAGCAACGTGGTCTTT |  |
| snc1-F(qPCR) | CCTCGAGTTGGTCTTGGTTG | Amplification of the *snc1* gene for qPCR |
| snc1-R(qPCR) | CTTGTCTTGCAGGACGTCCA |  |
| sso2-F(qPCR) | CATCACCAAGTACCAGACTG | Amplification of the *sso2* gene for qPCR |
| sso2-R(qPCR) | GTCTGTCGCTCTGCATCAGA |  |
| rho3-F(qPCR) | GACATGACAATCGAGCTCTG | Amplification of the *rho3* gene for qPCR |
| rho3-R(qPCR) | GATTGGTCAGCTTCGTATCC |  |
